# SnakeLines: integrated set of computational pipelines for sequencing reads

**DOI:** 10.1515/jib-2022-0059

**Published:** 2023-08-21

**Authors:** Jaroslav Budiš, Werner Krampl, Marcel Kucharík, Rastislav Hekel, Adrián Goga, Jozef Sitarčík, Michal Lichvár, Dávid Smol’ak, Miroslav Böhmer, Andrej Baláž, František Ďuriš, Juraj Gazdarica, Katarína Šoltys, Ján Turňa, Ján Radvánszky, Tomáš Szemes

**Affiliations:** Geneton Ltd., 841 04 Bratislava, Slovakia; Slovak Centre of Scientific and Technical Information, 811 04 Bratislava, Slovakia; Comenius University Science Park, 841 04 Bratislava, Slovakia; Department of Computer Science, Faculty of Mathematics, Physics and Informatics, Comenius University, 841 04 Bratislava, Slovakia; Department of Applied Informatics, Faculty of Mathematics, Physics and Informatics, Comenius University, 841 04 Bratislava, Slovakia; Department of Molecular Biology, Faculty of Natural Sciences, Comenius University, 841 04 Bratislava, Slovakia; Institute of Clinical and Translational Research, Biomedical Research Center, Slovak Academy of Sciences, 845 05 Bratislava, Slovakia

**Keywords:** computational pipeline, framework, massively parallel sequencing, reproducibility, virtual environment

## Abstract

With the rapid growth of massively parallel sequencing technologies, still more laboratories are utilising sequenced DNA fragments for genomic analyses. Interpretation of sequencing data is, however, strongly dependent on bioinformatics processing, which is often too demanding for clinicians and researchers without a computational background. Another problem represents the reproducibility of computational analyses across separated computational centres with inconsistent versions of installed libraries and bioinformatics tools. We propose an easily extensible set of computational pipelines, called SnakeLines, for processing sequencing reads; including mapping, assembly, variant calling, viral identification, transcriptomics, and metagenomics analysis. Individual steps of an analysis, along with methods and their parameters can be readily modified in a single configuration file. Provided pipelines are embedded in virtual environments that ensure isolation of required resources from the host operating system, rapid deployment, and reproducibility of analysis across different Unix-based platforms. SnakeLines is a powerful framework for the automation of bioinformatics analyses, with emphasis on a simple set-up, modifications, extensibility, and reproducibility. The framework is already routinely used in various research projects and their applications, especially in the Slovak national surveillance of SARS-CoV-2.

## 1 Background

Massively parallel sequencing (MPS) technologies have revolutionised not only research in molecular biology but also several clinical fields associated with genomic analyses. The rapid increase of genomic data has brought new challenges, mainly in transforming raw sequencing data into results interpretable by researchers and clinicians. Besides computational challenges, operatives must deal with a wide spectrum of available bioinformatics tools that are typically connected in computational pipelines. Development and testing of pipelines usually take a considerable amount of time and the process is prone to errors that are difficult to identify from the output files alone. Another problem is to ensure the reproducibility of the analysis across separated computational centres or distinct platforms with inconsistent software versions [1].

Several systems for the management of pipelines have been described and released to handle complex processing steps associated with MPS data [2]. All of these have stronger and weaker sides. For example, frameworks based on graphical interfaces [3, 4] are suitable for researchers without a strong computational background. On the other hand, frameworks based on the command-line interface (CLI) are more flexible, and so are typically preferred by bioinformaticians [5–7]. Lately, the CLI-based Snakemake workflow engine [8] gained a lot of attention, leading to several bioinformatic pipelines for various domains, such as metagenomics [9], variant calling [10], transcriptomics [11–13] and other epigenomics data [14, 15]. Although these pipelines are usually not limited to a single domain, sequence centres with broader scope may use several of them, and so handle multiple installations with different approaches to external dependencies, configuration, and execution (Table 1).

**Table 1:** Comparison of the selected Snakemake-based frameworks for bioinformatics analysis.

Framework	SnakeLines	MetaMeta	Sequana	VIPER	NGS-pipe	hppRNA	SnakeChunks	snakePipes
**Technical aspects**								
Language	Python R	Python	Python R	Python R Perl	Python R	Perl R	Python R	Python R
Installation of framework	Conda	Conda	Conda Pip Singularity	Download source	Download source	Installation script	Download source	Conda
Installation of dependencies	Automatic on-demand	Conda scripts	Manual	Conda scripts	Conda scripts	Installation script	Manual	Installation script
Configuration	YAML	YAML	YAML	YAML	JSON	Pipeline sources	YAML	YAML
Graphical interface	–	–	Yes	–	–	–	–	–
**Implemented pipelines**								
Assembly	Yes	–	Yes	–	–	–	–	–
Variant calling	Yes	–	Yes	–	Yes	–	–	–
Metagenomics	Yes	Yes	–	–	–	–	–	–
Transcriptomics	Yes	–	Yes	Yes	Yes	Yes	Yes	Yes
Viral identification	Yes	Yes	–	–	–	–	–	–
CNV detection	–	–	Yes	–	Yes	–	–	–
Chip-seq	–	–	–	–	–	–	Yes	Yes
Comprehensive epigenetics	–	–	–	–	–	–	–	Yes

We propose a set of Snakemake pipelines for a wider spectrum of bioinformatics analyses, called SnakeLines. The framework is designed to be easily extensible and adjustable with a single user-defined configuration file with an emphasis on the rapid deployment of required software and reproducibility of computational analysis. Although the pipelines were primarily developed for paired-end Illumina reads, they can be readily extended with the single-end Illumina and Nanopore specific tools to be applied to a wider set of sequencing technologies.

The open-source code of the proposed methods, together with test data, is freely available for non-commercial users at https://github.com/jbudis/snakelines along with Anaconda repository https://anaconda.org/bioconda/snakelines for rapid set-up and installation. Description of implemented pipelines, as well as for instructions for installation, running, and extending the framework, are accessible from the online documentation https://snakelines.readthedocs.io/.

## 2 Materials and methods

### 2.1 Snakemake framework

SnakeLines pipelines are compiled and executed by the Snakemake workflow engine [8], a widely-used tool for automating data analysis workflows, with a particular emphasis on bioinformatics pipelines.

Snakemake was designed to facilitate the development of reproducible, scalable data analyses. Its primary function is to provide a structured and automated approach to managing complex workflows, thereby streamlining the data analysis process and promoting efficient resource utilization.

The Snakemake framework operates by allowing users to define a series of rules that specify how data should be processed and output files generated. These rules can be written in various programming languages, including Python, R, and Shell scripts, thereby providing users with the flexibility to choose the language that best suits their needs. Subsequently, Snakemake constructs a Directed Acyclic Graph (DAG) that depicts the dependencies between the rules and input and output files.

The DAG serves as the blueprint for Snakemake’s workflow management process, enabling the system to execute the rules automatically and efficiently in parallel. The parallel execution of rules is of particular importance for processing large datasets, as it allows for the utilization of available computational resources in an optimized manner.

### 2.2 SnakeLines as an extension of the Snakemake framework

**Figure 1:**
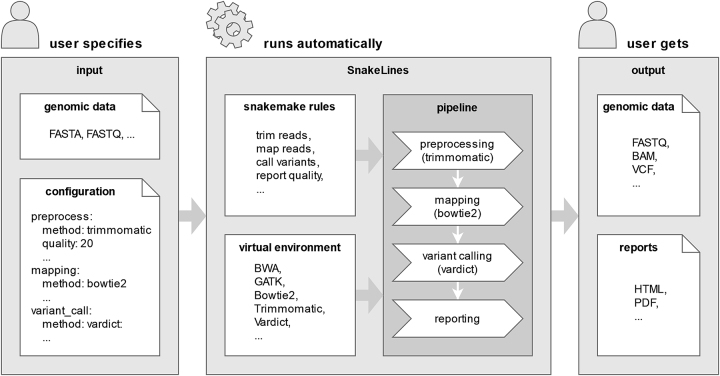
Standard execution of a SnakeLines pipeline: The user supplies the configuration file and genomic data originating from a sequencing run together with the required set of pipeline-specific files, such as a reference genome. Based on the configuration, SnakeLines identifies required Snakemake rules, bioinformatics tools, and their parameters. The exact steps of the computational pipeline are dynamically assembled and automatically executed in virtual environments using the Snakemake workflow engine. The output of the pipeline is a set of generated genomic files and a set of associated quality reports.

The SnakeLines framework extends the Snakemake engine with three main components: (1) the set of configurable rule templates for commonly used bioinformatics tools; (2) the set of custom Python scripts that build up the Snakemake pipeline from the rule templates based on the user-defined configuration file; (3) and the definition of virtual environments with all bioinformatics tools required for the execution of the pipeline (Figure 1).

At first, the SnakeLines provides a wide range of Snakemake rules that represent atomic operations of its pipelines, such as trimming of reads, mapping to reference sequences, or variant calling. Such rules are defined by mandatory input files, generated output files, and source code of operations that transform the input files into the output files. Dependencies between rules are automatically determined by Snakemake, defining a succession of operations that generates requested output files.

Since SnakeLines pipelines are executed with standard Snakemake calls, users may utilise its generous set of extended features, such as visualisation, monitoring, and parallel execution of pipelines that can be distributed over several computational nodes. SnakeLines adds extra functionality that allows simple set-up and modification of a pipeline from the single configuration file; including parameterization of used bioinformatics tools, their replacement with provided or custom alternatives, and omission or addition of requested processing steps. The user has overall information on all operations, as well as executed tools and their parameters. All required tools are automatically set up into isolated virtual environments to avoid common problems with their installation and inconsistent dependencies. SnakeLines uses Conda package repositories since they represent the most extensive source of bioinformatics packages from various language ecosystems used in the field [16]. This approach ensures the reproducibility of the analysis across different computational centres since all tools are installed in the same predefined versions.

### 2.3 Configuration of pipelines

Each SnakeLines pipeline is entirely defined by its configuration file in the YAML format. The user only has to supply a minimal set of input files for a pipeline execution; typically sequence reads (FASTQ format) and a reference genome (FASTA format). All reference indices required for an analysis are generated automatically during the pipeline execution. Selected output files and quality reports for downstream interpretation are aggregated into a single directory that may be easily exported and shared. The user may configure to be alerted at the end of a pipeline execution by an email message.

Each of the pipelines comes with a quality report, where available, and with a report summarising what was done and what are the results. These reports together with the most important output files are automatically copied at the end of a successful analysis into a user-supplied report directory.

**Figure 2:**
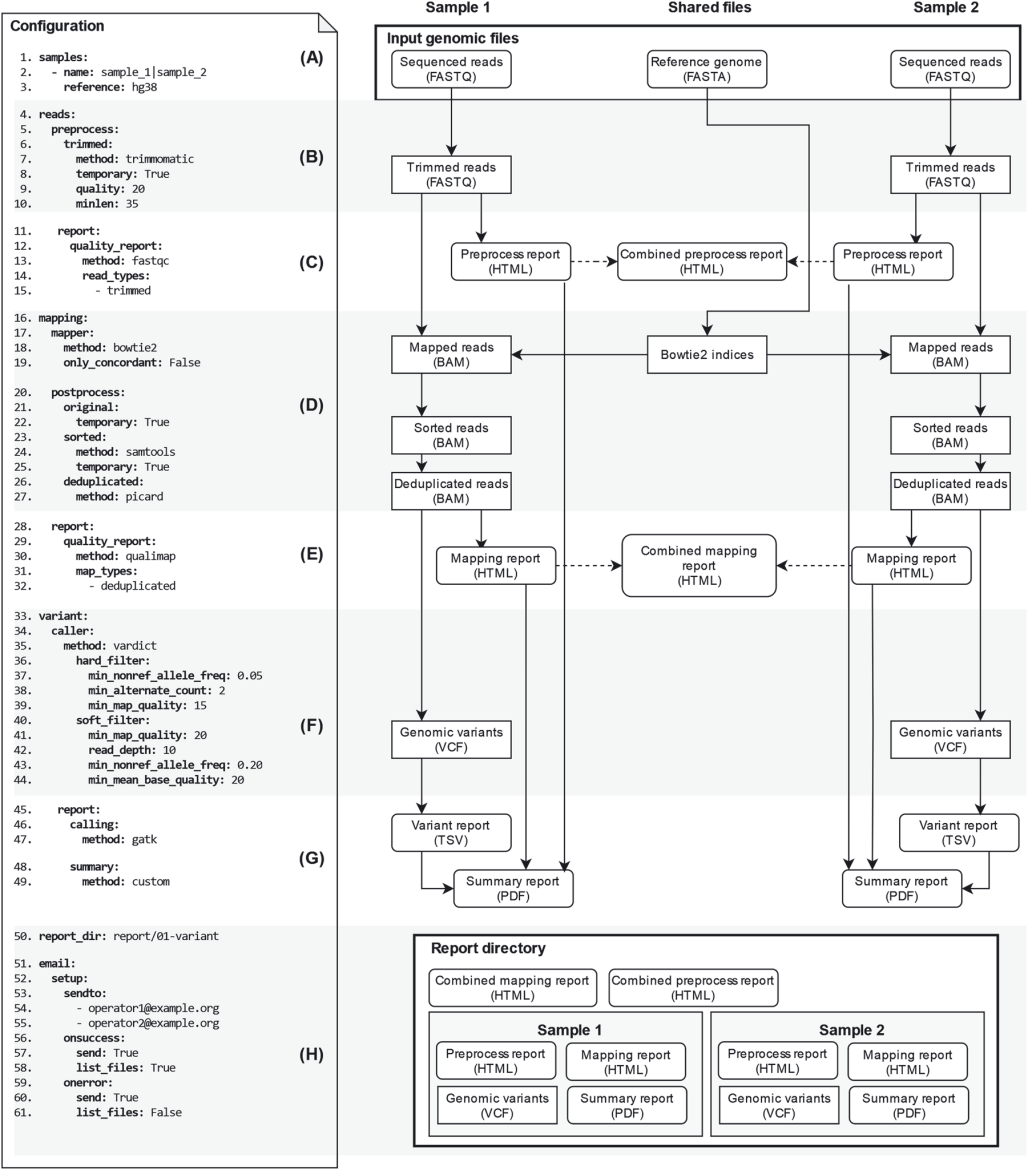
Basic variant calling pipeline constructed from the user-supplied configuration: (A) SnakeLines runs an analysis on specified FASTQ files (columns Sample 1, Sample 2). Each block of configuration (B)–(G) represents a set of SnakeLines rules that are automatically assembled into computational pipelines using the Snakemake workflow engine. The steps are gradually executed according to the generated workflow. (H) Essential output files are copied to the specified directory at the end of the analysis and users are notified by email messages.

The pipelines are processed in one of the three modes of operation, according to the source of sequenced reads: paired-end reads from Illumina sequencers, single-end reads from Nanopore sequencers and Illumina reads processed in single-end mode (Figure 2A). In the case of single-end read sequencing, several tools need to be properly set up to comply with the sequencing technology. For this purpose, an additional key ‘platform’ has to be supplemented with one of the values {illumina, nanopore}, so that SnakeLines loads appropriate versions of the required tools.

Descriptions of implemented pipelines, as well as instructions for installation, running, and extending the framework, are accessible from the online documentation https://snakelines.readthedocs.io/. Moreover, the documentation of a new rule is automatically gathered and deployed when this rule is included in SnakeLines.

## 3 Results

### 3.1 Implemented pipelines

We implemented several computational pipelines along with examples of input data for the rapid set up of the SnakeLines framework (Table 1). The pipelines reflect our experiences with bioinformatics processing, such as variant calling [17–19], *de novo* assembly [20], metagenomics [21–23], transcriptomics [24, 25] and applications in clinical diagnostics [26–28].

User-supplied sequenced reads are typically preprocessed at first to eliminate sequencing artefacts that may bias downstream analyses. The user may combine several implemented steps: trimming of low-quality ends [29], removal of duplicated fragments [30], filtering of reads from known hosts or contamination sources [31]. Pre-defined numbers of reads from each sample may be selected to avoid variability caused by uneven sequencing depth [32]. Finally, paired-end reads may be merged into singleton fragments based on their sequence overlap [33]. The effect of each step may be examined in HTML reports that are automatically generated using standard reporting tools [34, 35].

Preprocessed reads are passed to a downstream analysis that is chosen by the user according to the biological question to answer. Assembly of reads into contigs [36], for instance, can be chosen for novel organisms and customised for specifics of bacterial [37], metagenomic, transcriptomic, or plasmid-based biological material [38]. The quality of assembled contigs may be assessed using summary reports [39] or visual inspection of *de novo* assembly plots [40]. Contigs may be further annotated and reviewed in filterable and sortable HTML table with attributes, such as contig length, complexity, GC content of its sequence, homologous sequences in reference databases [41], and sequence similarity with viral genomes [42].

Mapping of the reads to a known genomic reference may be also customised according to the specifics of different types of sequenced material; including whole-genome or targeted sequencing [31, 43, 44], RNA transcripts [45], or bisulfite-treated DNA used in the analysis of methylation patterns [46]. All required indices are built automatically from provided reference sequences. Alternatively, the user may supply a list of accession ids and reference sequences will be automatically downloaded from the Genbank database (www.ncbi.nlm.nih.gov/genbank/), optionally followed by multiple alignments of the sequences [47] visualised in an interactive viewer [48], or as a phylogenetic tree [49]. SnakeLines also supports variant calling [50–53], as well as thorough classification of DNA fragments originating from a single target gene that are commonly used to study microbial communities [54–56] and identification of reads from viral genomes [57]. Generated reports are customised according to the type of input material. Besides summary tables and mapping reports for standard genomic material [35], SnakeLines provides specific bar plots and hierarchical pie plots [58] to visually assess the composition of microbial communities. Transcription profiles of RNA-Seq samples can be compared based on principal component analysis (PCA) reduced vectors [59]. Transcripts with a significant change in expression between various experimental conditions are identified and summarised in report tables [60].

### 3.2 Case study: variant calling

The identification of genomic variation in sequenced reads is a well-studied problem with a wide range of applications [61]. We chose, therefore, a basic variant calling pipeline to describe the fundamental concepts of the SnakeLines framework and how it can be customised through the single configuration file (Figure 2).

At first, the user declares a set of analysed samples (Sample 1 and Sample 2 in Figure 2A) along with the genomic reference of the sequenced organism (hg38). SnakeLines supports flexible declarations through regular expressions that allow to enumerate names of samples, choose them by pattern, or simply analyse all present samples. The user may also define several sets of samples with different references in a single configuration. SnakeLines checks the presence of the required files in the predefined directories and proceeds with the assembly of the pipeline.

The variant calling pipeline can be divided into three major steps; elimination of sequencing artefacts from sequenced reads (Figure 2B), mapping reads to a reference sequence (Figure 2D), and identification of variation in mapped reads (Figure 2F). Each step is recorded in the configuration to obtain a comprehensive overview of individual analysis steps, the tools used, and their parameters. In such a modular architecture, removing the block corresponding to the variant identification step (Figure 2F and G) would effectively reduce the pipeline to the preprocessing and the mapping step. Conversely, adding a block of configuration for other types of analysis, for example, a viral identification would lead to more complex analysis with additional reported files.

Processing steps in standard bioinformatics pipelines have typically well-defined and standardised types of input and output files. For example, mapping transforms sequenced reads in FASTQ format to mapped reads stored in BAM format, while requiring a reference genome in FASTA format. Similarly, variant calling transforms a BAM file into a VCF file. SnakeLines selects a rule that implements required transformation according to the ‘method:’ attribute that is specified for each processing step of the analysis separately (Figure 2, lines 10, 16, 21, 27, 30, 33, 38, 50, 52). In that manner, changing implementation can be easily done by replacing the value of the attribute. For example, the Bowtie2 and the BWA mapper can be readably switched by changing the ‘method: bowtie2’ to the ‘method: bwa’ (Figure 2D, line 21). In the case of a novel tool that is not already bundled in the set of SnakeLines rules, the user must first supply its Snakemake rule template with the same minimal set of inputs and outputs as its alternatives. This rule template must be placed into the same directory as other rules for that specific purpose. Also, the name of the rule template file must match its name, otherwise, SnakeLines would not be able to match the rule to the configuration.

The effect of individual steps may be examined in quality control reports that are stored in human-readable formats, such as HTML or PDF. Again, output reports can be customised, extended, or suppressed in the corresponding configuration blocks (Figure 2C, E, and G). The user may, for example, choose to generate an additional quality report for the original sequenced reads by an additional item ‘-original’ in the list of reported read types (Figure 2C, line 17). Reports are aggregated together into combined reports [62] to mitigate laborious inspection of numerous report files generated for each sample separately (Figure 2, dashed arrows).

SnakeLines also supports chaining of transformations for steps that produce the same types of files as types of their inputs. This is particularly useful for the post-process of generated files, for example, to sort a BAM file and then mark duplicated fragments (Figure 2D). Transformations are executed in the order declared in the configuration file. The BAM file generated in the last step of the chain (deduplication) is passed to the subsequent variant calling step. To avoid excessive amounts of stored data, the user may choose to remove outputs of these transformations by declaring the ‘temporary: True’ attribute (Figure 2, lines 11, 25, 28). Marked BAM files would be removed when no longer needed for further steps of the pipeline.

Bioinformatics pipeline typically generates numerous, mostly auxiliary files, while only a handful of them are typically used for assessing the quality of individual processing steps and interpretation of findings. Essential outputs, quality reports, and files required for reproduction (configuration file and SnakeLines version) are therefore copied automatically to the specified report directory at the end of the analysis (Figure 2H, line 53). Finally, specified users (Figure 2H, line 56) are notified by an email message that can also be sent in case of a failed analysis (Figure 2H, line 62).

**Figure 3:**
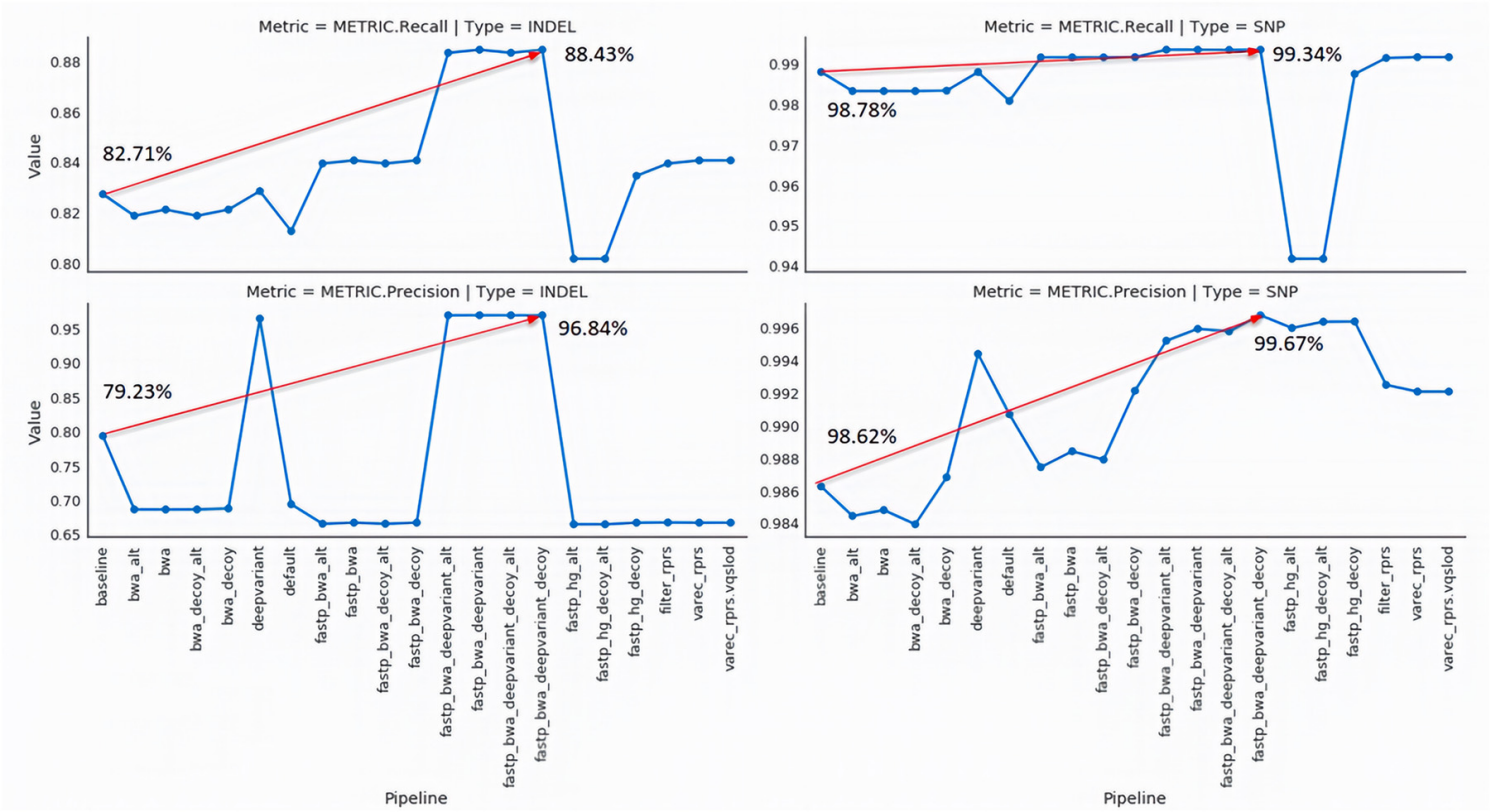
Benchmarking variant calling pipelines: SnakeLines facilitated the comparison between numerous configurations (only a handful shown for clarity) of variant calling pipelines for single nucleotide variants (right column) and small insertions and deletions (left column). The recall (top row) and precision (bottom row) have been considerably improved (arrows) between the initial (baseline) and the best performing configuration (fastp_bwa_deepvariant_decoy).

### 3.3 Case study: benchmarking variant calling pipelines

We utilised SnakeLines to find the best performing set of computational steps for variant calling pipeline on the human genome, namely, preprocessing (Figure 2B), mapping (Figure 2D), and variant calling itself (Figure 2F) The configuration-based approach to the set-up and parametrization of the pipeline enabled and greatly eased rapid testing of various pipelines (Figure 3). With the simple adjustments in SnakeLines configuration, we were able to test multiple versions of the human genome (Figure 2A), different computational tools, and parameters. Each configured pipeline ran separately and produced a set of detected variants, along with a report manifest with the precise information of the version of the SnakeLines and the associated configuration, allowing exact reproduction of the results.

The variant calls produced by SnakeLines from each pipeline configuration were compared to the high-confidence reference call set provided by Genome in a Bottle (GIAB) Consortium [63] outside of the SnakeLines framework. The comparison was performed according to the best GIAB practices [64] with the tool hap.py [65]. The default pipeline acted as a baseline and comprised Trimmomatic [29], Bowtie2 [31], and GATK HaplotypeCaller [31, 66]. We identified the optimal pipeline configuration considering precision and recall metrics on both SNVs and INDELs. This configuration consisted of GRCh38 human reference genome with decoy sequences and without alternative sequences, fastp [67] read preprocessor, BWA-MEM mapper [43], and DeepVariant caller [43, 52].

### 3.4 Variant calling pipeline for the SARS-CoV-2 national surveillance in Slovakia

The SnakeLines framework allowed us to easily adjust the variant calling pipeline to meet the specific challenges of amplicon-based NGS sequencing of SARS-CoV-2 genomes [68]. The pipeline has become a key part of the national COVID surveillance in Slovakia, now deployed in the two national computational infrastructures; the Comenius University Science Park and the Slovak Centre of Scientific and Technical Information, with more than 8000 samples analysed since the start of the national sequencing (March 2021–December 2021).

**Figure 4:**
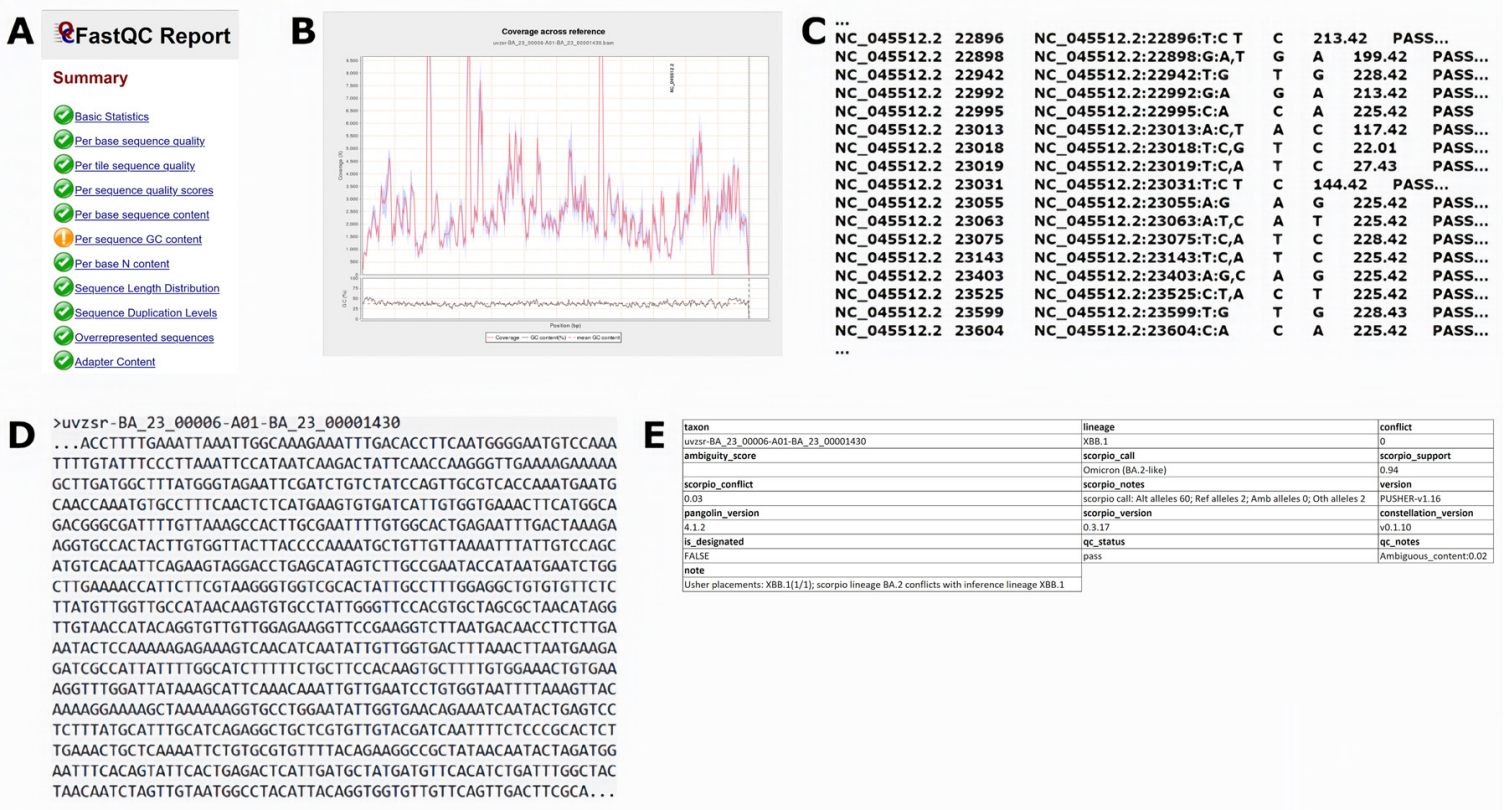
Selected reports generated by the Variant calling pipeline for the Sars-CoV-2 national surveillance in Slovakia, implemented in SnakeLines: (A) essential quality control metrics of raw sequencing reads, generated by FastQC [34]; (B) mapping coverage along the reference genome, generated by Qualimap BamQC [35]. (C) The consensus sequence of the analysed Sars-CoV-2 virus, shortened for clarity; (D) phylogenetic assignment of the sequenced virus, generated by the Pangolin [71]; (E) detected genomic variants in VCF format, shortened for clarity.

Several design changes needed to be performed in order to compensate for the issues and artefacts of the Illumina COVIDSeq Test sequencing protocol, including the removal of the ARTIC sequencing primers by the Cutadapt tool [69], and adjust mapping and variant calling tools, and their parameters, to improve the calling accuracy. These changes were facilitated by the simplistic nature of configuration files, in which the SnakeLines pipelines are defined. The rich variety of generated reports (FastQC, BamQC, MultiQC) allows detailed monitoring of sequencing data and early detection of sequencing artefacts. An example of these reports can be seen in Figure 4.

## 4 Discussion

SnakeLines is a powerful framework with a set of ready-to-use computational pipelines for several commonly used types of bioinformatics analyses. The configuration has been designed to provide a comprehensive view of all execution steps with emphasis on readability and flexibility in their adjustment and extension. Required bioinformatics tools are installed automatically into isolated virtual environments, which enable the rapid set-up of pipelines on fresh systems and also reproducibility of the analysis across different Unix-based platforms. Due to the powerful features of the underlying Snakemake engine, the execution of pipelines can be easily scaled from a single computer to distributed computational centres.

The SnakeLines framework aims to find the best compromise between easy-to-use graphical workflow managers and the flexibility of command-line-based solutions that require users with a computational background. Although the framework lacks a rich graphical interface, the configuration can be easily handled in any text editor application. Moreover, the basic text format may simplify the set-up of analysis through an external laboratory management system. Laboratory operator has complete control over individual processing steps, as well as implemented tools and their parameters. Although the SnakeLines does not yet provide the broad range of supported bioinformatics pipelines of well-established frameworks, such as Galaxy, the flexible architecture allows to include other tools and processing steps easily, and so have a great potential for further extensions to keep pace with the fast-moving field of nucleic acids analysis.

The presented framework has already become the inherent part of our data processing centre [22, 25, 70], and so would be further improved and extended with new bioinformatics tools and data analysis pipelines. The solution has been implemented on the two national computational infrastructures as a key part of the national COVID surveillance in Slovakia with more than 8000 samples analysed since the start of the national sequencing (March 2021–December 2021). Although the SnakeLines pipelines have been primarily designed and refined for the paired-end next-generation sequencing reads, the framework allows for readable incorporation of single-end or the third generation sequencing tools. We thus see great potential for a wide use of the framework across other research groups, due to its broad focus, simplicity, and rapid set up of required tools and dependencies.
